# “That’s just like, your opinion, man”: the illusory truth effect on opinions

**DOI:** 10.1007/s00426-023-01845-5

**Published:** 2023-06-10

**Authors:** Paul Riesthuis, Josh Woods

**Affiliations:** 1 Leuven Institute of Criminology, KU Leuven, Leuven, Belgium; 2 Faculty of Psychology and Neuroscience, Maastricht University, Maastricht, Netherlands; 3 Faculty of Psychology, Grand View University, Des Moines, IA USA

## Abstract

With the expanse of technology, people are constantly exposed to an abundance of information. Of vital importance is to understand how people assess the truthfulness of such information. One indicator of perceived truthfulness seems to be whether it is repeated. That is, people tend to perceive repeated information, regardless of its veracity, as more truthful than new information, also known as the illusory truth effect. In the present study, we examined whether such effect is also observed for opinions and whether the manner in which the information is encoded influenced the illusory truth effect. Across three experiments, participants (*n* = 552) were presented with a list of true information, misinformation, general opinion, and/or social–political opinion statements. First, participants were either instructed to indicate whether the presented statement was a fact or opinion based on its syntax structure (Exp. 1 & 2) or assign each statement to a topic category (Exp. 3). Subsequently, participants rated the truthfulness of various new and repeated statements. Results showed that repeated information, regardless of the type of information, received higher subjective truth ratings when participants simply encoded them by assigning each statement to a topic. However, when general and social–political opinions were encoded as an opinion, we found no evidence of such effect. Moreover, we found a reversed illusory truth effect for general opinion statements when only considering information that was encoded as an opinion. These findings suggest that how information is encoded plays a crucial role in evaluating truth.

Through the constant exposure of information on social media platforms such as Twitter, it has become increasingly important for people to assess what information is trustworthy and what should be discarded. Especially, since it has recently been shown that fake news is shared more often than truthful information (Vosoughi et al., 2018) and opinions are often believed to be widely shared by others and, therefore, resistant to change (Leviston et al., 2013; Lewandowsky et al., 2017). As platforms for facts, misinformation, and opinions are expanding, how do we determine truthfulness in the web of information? One indicator that influences people’s judgment regarding the accuracy of information is repetition. That is, repeated information tends to be regarded as more truthful than new statements (Brashier & Marsh, 2020a). However, does this effect remain for opinions as well? Moreover, does the manner in which information is initially encoded alter judgments of truthfulness? These two questions provided the primary aim of the three experiments.

## Illusory truth effect

Hasher and colleagues (1977) were one of the first to examine whether repeated information is perceived as more valid than new information. In their study, they instructed participants to judge the validity of true (e.g., “In Malaysia, if a man goes to jail for being drunk, his wife goes too”) and false statements (e.g., “Divorce is found only in technically advanced societies”). Then, two and four weeks later, participants were asked once again to rate the validity of true and false statements. Of these statements, some were previously presented while others were new. They found that repeated true and false statements were perceived as being more valid compared with new statements—an effect better known as the *illusory truth effect* (Brashier & Marsh, 2020b). In other words, repeating information led participants to believe that such information was more true than new information, even if factually incorrect.

Follow-up studies demonstrated the robustness of the illusory truth effect as it has been found for trivia statements (Bacon, 1979), COVID-19-related information (Unkelbach & Speckmann, 2021), fake news headlines (Pennycook et al., 2018), and even when people know the information is incorrect (Fazio et al., 2015). Apart from the robust effect of repetition on subjective truth ratings for factually true or false information, Arkes and colleagues (1989) examined whether the effect remained for social–political opinions (e.g., “Competition in schools is not good for young children”). To examine this, they used a similar procedure as Hasher and colleagues (1977) wherein participants had to rate the truthfulness of true, false, and social–political opinion statements. Immediately afterward, participants were again instructed to rate the truthfulness of the various statements, of which some were rated previously and some were new. Even though the authors concluded to have found the expected illusory truth effect pattern for social–political opinions (see Exhibit 4 of Arkes et al., 1989), their results were inconclusive. Specifically, Arkes and colleagues (1989, p. 85) stated “This interaction only approached significance, *F*(2, 194) = 2.73, *p* < 0.07. We nevertheless display it in Exhibit 4 to show that for all three types of statement—actually true, actually false, and opinions—the repeated statements rose in rated validity compared to their non-repeated counterparts.” Unfortunately, no further statistical analyses were conducted that examined the illusory truth effect separately for each type of statement. However, as approximately 55 articles^1^ cite Arkes and colleagues (1989) as evidence in favor of the illusory truth effect for opinions, we attempted to replicate it (Exp. 2 & 3) and examined the robustness by assessing whether such effect remained for general opinion statements (Exp. 1–3). That is, because beliefs about social–political opinions can be heavily influenced by personal social–political ideologies (Pennycook & Rand, 2021), we first examined whether the illusory truth effect remained for general opinion statements without social–political influence. Hence, we conducted three experiments to systematically examine the illusory truth effect for opinions.

Although the illusory truth effect is a robust phenomenon, one potential boundary condition can be the plausibility of statements (Pennycook et al., 2018). In other words, it seems the illusory truth effect is not observed when using implausible statements (e.g., “The Earth is a perfect square”). However, this may only occur for extreme implausible statements because previous research has shown that the illusory truth effect remained for false statements, even when participants indicated the correct answer afterwards (Fazio et al., 2015). This suggests that the manner in which information is encoded affects the illusory truth effect (Brashier et al., 2020a; Fazio et al., 2015; Pennycook et al., 2015, 2018). More specifically, when participants encode a statement and can identify the implausibility or inaccuracy, they might not perceive repeated statements as being more subjectively true than new statements. Moreover, previous research showed that the illusory truth effect is not observed when information is presented by an untrustworthy voice or when participants acted as fact checkers (Begg et al., 1992, experiment 4; Brashier et al., 2020a). To assess this further, we additionally examined whether the way in which information is encoded alters the illusory truth effect for true information, misinformation and opinion statements.

## Theoretical frameworks

There are several explanations for the illusory truth effect (Unkelbach et al., 2019). One prominent explanation is the processing fluency account (Reber & Schwarz, 1999) which posits that people regard information as more truthful when it is processed more fluently. A prime example was given by Reber and Schwarz (1999) who found that information presented in easy to read font was rated as being more truthful compared with information in difficult to read font. Following this logic, the idea is that repeated information is processed more fluently than new information because it feels more familiar (Arkes et al., 1989) and is recognized (Bacon, 1979), leading to higher subjective truth ratings. Additionally, it is argued that the mere repetition of information leads to higher subjective truth ratings because it is more likely that the single account of the truth is more often repeated than the various alternative (incorrect) versions (Brashier & Marsh, 2020b).

Another explanation for why repeated information is processed more fluently than new information is given by the referential theory of Unkelbach and Rom (2017). This theory postulates that repeated information is processed more fluently because it enhances the coherency between elements of information which serves as an indicator of subjective truth (Unkelbach et al., 2019). Specifically, truth judgments are informed by the specific meanings of individual elements (e.g., words) in a statement and how coherent these individual elements are with one another based on previous knowledge (Unkelbach & Rom, 2017). For instance, when people encode the statement “The pan-American highway is the longest road in the world” it is possible that the elements “pan-American highway” and “road” are perceived as coherent based on previous knowledge. However, new elements, such as “longest” and “in the world”, may not have been associated to “pan-American highway” and “road”. The referential theory posits that the more coherent elements within a statement, the more easily such information is processed, leading to higher subjective truth ratings. One proposed process that increases such coherency is repetition, especially with relatively unknown statements. Therefore, according to the referential theory, statements containing incoherent elements do not lead to the illusory truth effect, even when repeated.

The main difference between the processing fluency account and the referential theory is that the latter attempts to explain why fluently processed information is judged with higher truth ratings. To date, both theories provide evidence that repeated information leads to higher truth ratings due to increasing the coherency between elements and/or the fluency of information processing (e.g., Unkelback & Rom, 2017). An empirical question was whether coherency and/or processing fluency are disrupted for opinions wherein its subjective information might lower the perceived coherency and/or ease of processing, especially when encoded as an opinion.

## Experiment 1

The crux of Experiment 1 was to examine the illusory truth effect for true information, misinformation, and general opinion statements, when they are encoded as an opinion or fact based on its syntax structure. To do so, participants were first presented with various (i.e., true information, misinformation, and general opinion) relatively unknown statements and had to indicate whether a statement was a fact or an opinion based on its syntax structure (e.g., fact = “The zipper was invented by Elias Howe in 1851”; opinion = “Walter Hunt was a *brilliant* inventor who created the safety pin”). Syntax structure for true information and misinformation statements were characterized by verbs (e.g., “is”, “was”) that indicated factual information (e.g., names, years, and events) void of subjective information (e.g., “best”, “should”, “largest”). General opinion statements were transformed to closely resemble factual statements. That is, to have a highly controlled comparison, general opinion statements had similar verbs and factual information as in true information and misinformation statements but contained the aforementioned subjective information. Then, after a short distractor task, participants had to rate the truthfulness of previously presented and new statements. Because we used relatively unknown but plausible statements, we expected that repeating information, regardless of the type of statement (i.e., true information, misinformation, and general opinion), would lead to more coherency and enhance the processing fluency and therefore higher subjective truth ratings compared with new information. Including such general opinion statements will test the robustness of the illusory truth effect by examining whether it remains with varying stimuli as recommended by Henderson and colleagues (2021). Second, because opinions statements contained words indicating the subjectiveness (e.g., “best”), we expected the coherency between elements within the statement to be decreased. Because of this decreased coherency, we expected that statements that were repeated and encoded as facts by the participants during Part 1 (vs. opinions encoded as an opinion) would ease the processing of information and, in turn, lead to higher subjective truth ratings.

## Methods

### Participants

An a priori power analysis conducted for a two-tailed paired-sample t test in G*Power (Faul et al., 2009) with a power of 0.80, Cohen’s *d*_z_ = 0.21, and α = 0.05 indicated 180 participants were needed. We conducted the power analysis for two-tailed paired-sample t tests to have sufficient statistical power for our exploratory analyses (two-tailed) while also achieving sufficient statistical power for our confirmatory analyses (i.e., directional, one-tailed). Moreover, we performed a two-tailed paired-sample t test because the results of the illusory truth effect for opinions were inconclusive (Arkes et al., 1989). Our effect size was based on our smallest effect size of interest (SESOI) for which we provided a cost–benefit analysis (Lakens, 2014). That is, because framing and encoding information in the form of a fact (vs. opinion) is such a low-cost manipulation, we were interested in whether it can result in a 0.2 raw mean difference in subjective truth ratings on a 7-point Likert scale. Such low-cost manipulations can be easily repeated on, for example Twitter via retweets, meaning that the effects might accumulate (Funder & Ozer, 2019). Previous research has indeed shown that additional repetitions can increase the illusory truth effect (Hassan & Barber, 2021). Additionally, because we used ambiguous statements, a 0.2 increase in subjective truth ratings on a 7-point Likert scale can be crucial in whether participants deem the repeated information as the truth. Specifically, a 0.2 increase in subjective truth rating can push participants to judge an otherwise deemed untruthful or ambiguous statement (Likert score ≈ 4) toward the truthful side, especially when repeated multiple times. The cost of framing information in terms of a fact or an opinion is rather low and, therefore, a 0.2 increase in subjective truth can already be of interest. Moreover, the costs of simple instructions such as evaluating whether information is a fact or an opinion are also low. These simple instructions are of interest if they can increase people’s subjective truth rating for correct information or protect them from judging, for example, opinions as more truthful. Since the effects of framing and encoding information can apply to both true information, misinformation, and general opinion statements, we deemed a 0.2 raw mean difference as the SESOI for the illusory truth effect when analyzing all statement types together (true information, misinformation, and general opinion) but also when analyzing the type of statements separately. Based on previous research examining the illusory truth effect using similar Likert scale measures (Unkelbach & Speckmann, 2021), we calculated that a 0.2 raw mean difference in subjective truth ratings is equal to a Cohen’s *d*_*z*_ = 0.21.^2^ We recruited 182 participants from the United States of America via Amazon Mechanical Turk. MTurk participants were recruited by posting a Human Intelligence Task (HIT) named “How do we judge statements?” The requirements for MTurk participants were a HIT approval rate of 98% and more than 5000 HITs approved. Two attention checks were included in Part 1 and Part 2 of the experiment. Only one participant failed both attention checks and, as outlined in the preregistration, we excluded the data of this participant from the data analysis.^3^ In total, we remained with the data of 181 participants for the data analysis (*M*_age_ = 42.5, *SD*_age_ = 11.3, range = 23–76; 98 women, 82 men, 1 non-binary. Participants took on average 23.4 min (*SD* = 10.8; range = 11.2–95.1) to complete the study. All participants were financially compensated $2.00 for participation, regardless of whether they correctly answered the attention checks. 

The Institutional Review Board of Grand View University approved the study. Moreover, we preregistered the study on the Open Science Framework (OSF; https://osf.io/kxypn/?view_only=72825e53b53b4f2498d0641e4125beb8),^4^ and uploaded the raw data and R script (https://osf.io/u67kg/?view_only=20c8eeb39e884f81a4345629bcb4322d).

## Materials

We used a list of 99 statements successfully implemented in previous research which were shared by the second author (Hassan & Barber, 2021). Sixty-six of these statements were normed in previous research and it was shown that they were relatively unknown but plausible (Mutter et al., 1995). The remaining 33 statements of Hassan and Barber were found via online sources but were not normed. Their research team judged them as being relatively unknown but plausible. For our experiment, we transformed 33 of these statements into misinformation statements and another 33 into general opinion statements resulting in three types of statements: True information, misinformation, and general opinion. For instance, to create a misinformation statement, we adapted a true information statement like “The refrigerator was invented in 1748 by William Cullen” into “The refrigerator was invented in 1898 by Thomas Paine” and to create a general opinion statement we transformed it into “The refrigerator was the greatest invention of the 1700s”. We selected 27 critical items a priori that would be repeated which were used for the statistical analyses. The 27 critical items consisted of nine true information, nine misinformation, and nine general opinion statements (see Appendix A and preregistration). For Part 1 wherein participants had to indicate whether a statement was a fact or an opinion, we included 36 distractor statements of which 12 were true information, 12 misinformation, and 12 general opinion statements. In total, there were 63 statements for Part 1, of which 27 critical items and 36 distractor items. Distractor items were added to make sure that participants were not able to easily detect which statements were repeated during Part 2 of the study. Statements in Part 1 were presented completely at random for each participant. For Part 2 wherein participants rated the truthfulness of statements, we added 36 new statements of which 12 were true information, 12 misinformation, and 12 general opinion statements. To match the number of critical items, we chose a priori 27 control statements out of those 36 new statements. The 27 control statements consisted of nine statements for each type of statement (see Appendix A and preregistration). We chose 9 critical and 9 control statements for each type of stimuli (i.e., true information, misinformation, general opinions) to keep the amount of stimuli in Part 1 and Part 2 the same. Moreover, in line with recommendations for research with MTurk samples (Aguinis et al., 2021), we did not want to increase the number of critical and control statements to not overly burden the participants as it has been shown that longer experiments can lead to unreliable data.^5^ Hence, in total, there were also 63 statements for Part 2, of which 27 were repeated (i.e., critical items) and 36 were new (i.e., 27 control and 9 distractor statements). We added 9 distractor statements to have the same amount of stimuli during Part 1 and Part 2 which also served to prevent participants from detecting which statements were repeated. Statements in Part 2 were presented completely at random for each participant. Subjective truth ratings were given on a 7-point Likert scale (*1* = *not truthful, 7* = *very truthful*).

Last, we included two attention checks in the experiment. The first attention check (i.e., “The present city of Atlanta was originally named Terminus [To show that you have read this information, we would like you to answer “Opinion” on this page]”) was placed completely at random in Part 1 while the second attention check (i.e., “1 in 5,000 north Atlantic lobsters are born bright blue. [To show that you have read this information, we would like you to answer “1 (Not truthful)” on this page]”) was placed at random in Part 2. We used two attention checks to distinguish inattentive participants from simple mistakes (Abbey & Meloy, 2017). Moreover, following best practices for attention checks (Thomas & Clifford, 2017), we used different types of attention checks, which were in line with the experiment and fair in terms of clearly indicating the desired answer.

## Procedure and design

In the present experiment, we used a within-subject design. Participants were first instructed to read the informed consent. If they agreed to participate, they received the following instructions:

*“Previous research has revealed that high school students are not always able to distinguish between how fact and opinions are constructed. In this study, we are interested whether you are able to indicate whether a statement is a fact or an opinion based on its syntax structure. Hence, in the next phase, you will receive multiple statements and then we would like you to indicate whether it is a fact or an opinion.”*

After participants completed Part 1 of the experiment wherein they indicated whether a statement was a fact or an opinion, they engaged in a 5-min filler task (i.e., playing Tetris). Then, in Part 2 of the experiment, participants were given a list of 63 statements (27 critical repeated items, 27 control/new items, 9 distractor/new items) and were asked to indicate the truthfulness of each statement on a 7-point Likert scale (*1* = *not truthful, 7* = *very truthful*). Afterward, participants were thanked and debriefed.

## Results

### Statistical analyses

Based on visual inspection of the QQ plots, histograms, and boxplots, we found the data to be normally distributed. Hence, for all analyses, we conducted paired-sample t tests to examine the difference in subjective truth ratings for repeated versus new statements. Moreover, because we set a SESOI, we also ran equivalence tests using the two one-sided tests procedure (TOST; Lakens, 2017).^6^ Typically, in null hypothesis significance testing (NHST), it is examined whether the observed effects are statistically significantly (e.g., *p* < 0.05) different from zero. A limitation of the traditional NHST is that we can never establish that the true effect size of an observed phenomenon is precisely zero (i.e., no effect) and can only conclude that there was insufficient statistical power to detect an effect (Lakens, 2017). However, one possibility using the frequentist approach is to conduct equivalence tests wherein researchers can determine whether the observed effect is statistically equivalent, even if statistically significant. In other words, if the observed effects are statistically significant (i.e., 95% CI of the mean difference does not include 0) but also statistically equivalent (i.e., 90% Confidence Interval [CI] of the mean difference is within the lower and upper equivalence bounds), they are simply too small to care about. If a statistically significant effect is detected and the 90%CI includes the lower or upper equivalent bound, it means that the result is not statistically equivalent (one of the two one-sided tests has a *p* > 0.05) and could be a practically relevant effect based on the SESOI. This can be done by establishing, preferably, a priori the SESOI and its justification that indicates the threshold of meaningful effects for practical implications and/or theoretical matters (Lakens et al., 2018). Hence in our study, we used our SESOI (raw mean difference = 0.2) to establish the lower (Δ_*L*_) and upper (Δ_*U*_) bounds (i.e., equivalence bounds) to assess whether the observed effects were statistically equivalent and statistically different (see Lakens, 2017).

## Manipulation and attention checks

First, we examined whether participants successfully encoded the initial statements in Part 1 as either a fact or opinion based on the syntax structure. Participants correctly encoded, on average, 90.1% of true information statements as facts, 84.6% of misinformation statements as facts, and 82.3% of general opinion statements as opinions. A two-tailed test for equality of proportions showed that true information statements were not encoded statistically significantly better as a fact than misinformation statements, *X*^*2*^ (1) = 0.091, *p* = 0.76, 95% CI [− 0.14, 0.25]. This indicates that participants generally encoded the information as intended. However, we also performed exploratory analyses wherein we only included the statements that were correctly encoded as a fact or opinion in Part 1 (see Exploratory Analyses).

Examining the attention checks, we found two participants failed the first attention check and nine participants failed the second attention check. Only one participant failed both attention checks and, as preregistered, the data of these participants were excluded from the data analyses.

## Confirmatory analyses

### Repeated versus new statements

*Illusory Truth Effect*. We first examined whether there was an overall illusory truth effect across all statements and then scrutinized the illusory truth effect for each type of statement (true information, misinformation, and general opinion). A one-tailed paired-sample t test showed that repeated statements received higher subjective truth ratings (*M* = 4.50, *SD* = 0.94) compared with new statements (*M* = 4.31, *SD* = 0.89), *t *(180) = 4.59, *p* < 0.001, Cohen’s *d*_*z*_ = 0.34, 95% CI [0.22, ∞] (see Table 1). Additionally, the TOST procedure with raw equivalence bounds Δ_*L*_ = -0.20 and Δ_*U*_ = 0.20 showed a statistically significant result against the Δ_*L*_, *t* (180) = 9.29, *p* < 0.001, but did not find a statistically significant result against the Δ_*U*_, *t* (180) = − 0.11, *p* = 0.46. These results indicate that the illusory truth effect across all statements was statistically significant and not statistically equivalent.

**Table 1:** Descriptive Statistics of Subjective Truth Ratings for each Type of Statement of Experiment 1

Type of Statement	*N*	*M*	*SD*
All Statements			
Repeated	181	4.50	0.94
Repeated–correctly encoded	181	4.59	0.89
New–control	181	4.30	0.89
True Information			
Repeated	181	5.25	1.11
Repeated–correctly encoded	181	5.41	1.11
New–control	181	5.09	1.05
Misinformation			
Repeated	181	4.33	1.55
Repeated–correctly encoded	178	4.59	1.51
New–control	181	3.84	1.33
General opinion			
Repeated	181	3.93	1.13
Repeated–correctly encoded	181	3.78	1.12
New–control	181	3.99	1.07

*N* stands for sample size,* M* stands for mean, *SD* stands for standard deviation. Repeated statements were the 9 critical items chosen a priori that were presented during Part 1 and therefore repeated in Part 2. Repeated – Correctly Encoded statements included only critical items that were correctly encoded as a “fact” or “opinion” during Part 1. New – Control statements were control items that were only presented once during Part 2. Three participants did not correctly identify any misinformation statement as a fact based on its syntax structure and were removed from the analyses. Removing the participants did not alter the pattern of results.

*True Information*. A one-tailed paired-sample t test showed that repeated true information statements received higher subjective truth ratings (*M* = 5.25, *SD* = 1.11) compared with new statements (*M* = 5.09, *SD* = 1.05), *t* (180) = 2.68, *p* = 0.004, Cohen’s *d*_*z*_ = 0.20, 95%CI [0.08, ∞]. Additionally, the TOST procedure with raw equivalence bounds Δ_*L*_ = − 0.20 and Δ_*U*_ = 0.20 showed a statistically significant result against the Δ_*L*_, *t* (180) = 6.09, *p* < 0.001, but did not find a statistically significant result against the Δ_*U*_, *t* (180) = − 0.72, *p* = 0.24. These results show that the illusory truth effect for true information statements was statistically significant and not statistically equivalent.

*Misinformation*. A one-tailed paired-sample t test showed that repeated misinformation statements received higher subjective truth ratings (*M* = 4.33, *SD* = 1.55) compared with new statements (*M* = 3.84, *SD* = 1.33), *t* (180) = 6.51, *p* < 0.001, Cohen’s *d*_*z*_ = 0.49, 95%CI [0.36, ∞]. Moreover, the TOST procedure with equivalence bounds Δ_*L*_ = − 0.20 and Δ_*U*_ = 0.20 showed a statistically significant result against the Δ_*L*_, *t* (180) = 9.16, *p* < 0.001, but did not find a statistically significant result against the Δ_*U*_, *t* (180) = 3.86, *p* > 0.99. These results show that the illusory truth effect for misinformation statements was statistically significant and not statistically equivalent.

*General Opinion*. A one-tailed paired-sample t test did not find a statistically significant difference between subjective truth rating for general opinion statements that were repeated (*M* = 3.93, *SD* = 1.13) compared with new statements (*M* = 3.99, *SD* = 1.07), *t* (180) = -1.13, *p* = 0.87, Cohen’s *d*_*z*_ = -0.08, 95%CI [− 0.21, ∞]. Additionally, results from the TOST procedure with equivalence bounds Δ_*L*_ = − 0.20 and Δ_*U*_ = 0.20 showed statistically significant results against the Δ_*L*_, *t* (180) = 2.44, *p* = 0.008 and against the Δ_*U*_, *t* (180) = − 4.70, *p* < 0.001. These results show that the illusory truth effect for general opinions statements was not statistically significant and was statistically equivalent.

*Facts versus Opinions*. To examine whether the illusory truth effect is stronger for correctly encoded facts than correctly encoded general opinions, we compared the observed mean difference in subjective truth ratings between repeated and new statements for facts (i.e., true information and misinformation statements) and general opinion statements. A one-tailed paired-sample t test revealed that the illusory truth effect was enhanced for correctly encoded facts (*M* = 0.50, *SD* = 0.77) versus correctly encoded general opinions (*M* = − 0.21, *SD* = 0.91), *t* (180) = 7.93, *p* < 0.001, Cohen’s *d*_*z*_ = 0.59, 95% CI [0.46, ∞]. In other words, true information statements that were repeated led, on average, to an increase of 0.50 subjective truth ratings on a 7-point Likert scale (vs. new statements), while for opinion statements the subjective truth ratings decreased with 0.21 (Δ_illusory truth effect_ = 0.71). Additionally, results from the TOST procedure revealed using equivalence bounds of Δ_*L*_ = − 0.20 and Δ_*U*_ = 0.20 showed statistically significant results against the Δ_*L*_, *t* (180) = 10.17, *p* < 0.001 but not against the Δ_*U*_, *t* (180) = 5.68, *p* > 0.99. This indicates that the illusory truth effect was statistically significantly enhanced for facts compared with general opinion statements and this was not statistically equivalent.

## Exploratory analyses

Our manipulation check showed that participants did not encode all statements correctly as a fact or an opinion based on its syntax structure. In the exploratory analyses, we ran the same statistical analyses as described above but only with the statements that were correctly encoded as a fact or an opinion during Part 1 of the study. That is, for each participant, we excluded the data for true information and misinformation statements that were encoded as an opinion in Part 1, and excluded the data for opinion statements that were encoded as facts.

*Illusory Truth Effect*. A two-tailed paired-sample t test showed that correctly encoded repeated statements received higher subjective truth ratings (*M* = 4.59, *SD* = 0.89) compared with new statements (*M* = 4.31, *SD* = 0.89), *t* (180) = 6.29, *p* < 0.001, Cohen’s *d*_*z*_ = 0.47, 95%CI [0.31, 0.62]. Moreover, a TOST procedure with raw equivalence bounds of Δ_*L*_ = − 0.20 and Δ_*U*_ = 0.20 showed statistically significant results against the Δ_*L*_, *t* (180) = 10.64, *p* < 0.001 but not against the Δ_*U*_, *t* (180) = 1.94, *p* = 0.97. These results show that the illusory truth effect for correctly encoded statements was statistically significant and was not statistically equivalent.

*True Information*. A two-tailed paired-sample t test revealed that repeated true information statements that were correctly encoded as a fact during Part 1 had higher subjective truth ratings (*M* = 5.41, *SD* = 1.11) compared with new true information statements (*M* = 5.09, *SD* = 1.05), *t* (180) = 4.81, *p* < 0.001, Cohen’s *d*_*z*_ = 0.36, 95% CI [0.21, 0.51] (see Table 1). Moreover, a TOST procedure with raw equivalence bounds of Δ_*L*_ = − 0.20 and Δ_*U*_ = 0.20 showed statistically significant results against the Δ_*L*_, *t* (180) = 7.82, *p* < 0.001 but not against the Δ_*U*_, *t* (180) = 1.79, *p* = 0.96. These results show that the illusory truth effect for correctly encoded true information statements was statistically significant and was not statistically equivalent.

*Misinformation*.^7^ A two-tailed paired-sample *t* test revealed repeated misinformation statements that were correctly encoded as a fact during Part 1 had higher subjective truth ratings (*M* = 4.59, *SD* = 1.51) compared with new misinformation statements (*M* = 3.87, *SD* = 1.31), *t* (177) = 8.72, *p* < 0.001, Cohen’s *d*_*z*_ = 0.66, 95%CI [0.49, 0.82]. Moreover, a TOST procedure with raw equivalence bounds of Δ_*L*_ = .− 20 and Δ_*U*_ = 0.20 showed statistically significant results against the Δ_*L*_, *t* (177) = 11.13, *p* < 0.001 but not against the Δ_*U*_, *t* (177) = 6.31, *p* > 0.99. These results show that the illusory truth effect for correctly encoded true information statements was statistically significant and was not statistically equivalent.

*General Opinion*. A two-tailed paired-sample t test revealed repeated general opinion statements that were correctly encoded as an opinion during Part 1 had lower subjective truth ratings (*M* = 3.78, *SD* = 1.12) compared with new general opinion statements (*M* = 3.99, *SD* = 1.07), *t* (180) = − 3.07, *p* = 0.002, Cohen’s *d*_*z*_ = − 0.23, 95%CI [− 0.38, − 0.08]. Moreover, a TOST procedure with raw equivalence bounds of Δ_*L*_ = -0.20 and Δ_*U*_ = 0.20 did not show statistically significant results against the Δ_*L*_, *t* (180) = − 0.11, *p* = 0.54 but did find statistically significant results against the Δ_*U*_, *t* (180) = − 5.99, *p* < 0.001. These results show that the repeated correctly encoded general opinion statements received statistically significantly lower subjective truth ratings compared with new general opinion statements and this was not statistically equivalent.

## Enhanced illusory truth effect

As a second exploratory analysis, we compared the raw mean differences of the illusory truth effect for true information and misinformation statements when only taking into account those that were correctly classified as a fact based on its syntax structure against the stimuli in general (correctly and incorrectly classified information). This analysis can indicate whether the illusory truth effect was enhanced when true and misinformation statements were correctly classified as a fact.^8^

*True Information*. A two-tailed paired-sample t test also showed that the illusory truth effect was enhanced when only taking into account true information statements correctly encoded as a fact during Part 1 (*M* = 0.32, *SD* = 0.89) compared with true information statements in general (*M* = 0.16, *SD* = 0.79), *t* (180) = 4.55, *p* < 0.001, Cohen’s *d*_*z*_ = 0.34, 95%CI[0.19, 49]. Additionally, following the TOST procedure with raw equivalence bounds of Δ_*L*_ = − 0.20 and Δ_*U*_ = 0.20 showed statistically significant results against the Δ_*L*_, *t* (180) = 10.20, *p* < 0.001 but not against the Δ_*U*_, *t* (180) = − 1.09, *p* = 0.14. This showed that the illusory truth effect was statistically significantly stronger for true information statements correctly encoded compared with all true information statements and this was not statistically equivalent.

*Misinformation*. Moreover, a two-tailed paired-sample t test showed that the illusory truth effect was enhanced when only taking into account misinformation statements correctly encoded as a fact during Part 1 (*M* = 0.68, *SD* = 1.15) compared with misinformation statements in general (*M* = 0.49, *SD* = 1.01), *t* (180) = 3.76, *p* < 0.001, Cohen’s *d*_*z*_ = 0.28, 95% CI [0.13, 43]. The TOST procedure with raw equivalence bounds of Δ_*L*_ = − 0.20 and Δ_*U*_ = 0.20 revealed a statistically significant result against the Δ_*L*_, *t* (180) = 7.76, *p* < 0.001 but not against the Δ_*U*_, *t* (180) = − 0.240, *p* = 0.41. This showed that the illusory truth effect was statistically significantly stronger for misinformation statements correctly encoded compared with all true information statements and this was not statistically equivalent.

## Discussion

In experiment 1, we examined whether repeating true, misinformation and general opinion statements would be perceived as more truthful compared with new statements. In line with previous research (e.g., Dechêne et al., 2010) and our predictions, we found the typical illusory truth effect. However, when scrutinizing the illusory truth effect for each type of statement, we only found that repeated true information and misinformation statements led to higher subjective truth rating relative to new statements. Moreover, our results showed the illusory truth effect was stronger when only taking into account true information statements that were correctly encoded as a fact by the participants. These findings highlight the replicability of the illusory truth effect in different contexts and also lend support to the idea that it can be observed using a short delay between repetitions (Henderson et al., 2021). In fact, these results suggest that the illusory truth effect might be enhanced for true information and misinformation statements when they are encoded as a fact.

Interestingly, we failed to find evidence for the illusory truth effect for general opinion statements. In other words, our findings suggest that repeated general opinion statements that were correctly encoded as an opinion did not increase the perceived truthiness of such statements in comparison with new statements, but actually led to a reversed illusory truth effect. Specifically, repeated general opinion statements that were correctly encoded as an opinion scored lower on truthfulness compared with new general opinion statements. This is in contrast with the conclusions made by Arkes and colleagues (experiment 1, 1989). That is, they argued the illusory truth effect remained even for opinions, while our data did not show support for this and even indicated that the effect might be reversed for such statements. However, our general opinion statements differed from the stimuli of Arkes and colleagues (1989) as they used social–political opinions. It is possible that the illusory truth effect is found for social–political opinion statements.

In Experiment 2, we replicated Experiment 1 to examine the illusory truth effect for true information and general opinion statements but also for social–political opinion statements. We expected to again find the illusory truth effect for true information statements. We also predicted that repeated general and social–political opinion statements (correctly and incorrectly classified as an opinion) would not increase subjective truth ratings relative to new statements. Moreover, as seen in Experiment 1, we expected a reversed illusory truth effect for correctly encoded general and social–political opinions. That is, we expected that general and social–political opinions that are encoded as an opinion would receive lower subjective truthfulness ratings compared relative new statements. Additionally, based on our findings in Experiment 1, we predicted that the illusory truth effect would be enhanced when only considering true information statements that were encoded as a fact. We also predicted to observe a reversed illusory truth effect when only considering the general and social–political statements that were encoded as an opinion during Part 1.

## Experiment 2

Experiment 2 was identical to experiment 1, but we replaced the 33 misinformation statements with social–political statements.

## Methods

### Participants

We used the same a priori power analysis as in Experiment 1 which indicated that we needed 180 participants. In total, we recruited 186 participants from Amazon Mechanical Turk. MTurk participants were recruited by posting a hit named “How do we judge statements?” The requirements for Mturk participants were a HIT approval rate of 98% and more than 5000 HITs approved. As in Experiment 1, we had two attention checks in Part 1 and Part 2 of the experiment. One participant failed both attention checks, so the data of this participant for the data analysis was excluded. Hence, we had data from 185 participants for the statistical analyses (*M*_age_ = 40.8, *SD*_age_ = 12.6, range = 23–76; 104 men, 81 women). Participants took on average 24.4 min (*SD* = 9.3; range = 10.9–57.0) to complete the study. All participants were financially compensated $2.00 for participation, regardless of whether they correctly answered the attention checks.

The Institutional Review Board of Grand View University approved the study. Moreover, we also pre-registered Experiment 2 on the OSF (https://osf.io/v98d3/?view_only=111181c9cb6a42b2bcb4b8596982bd44), and uploaded the data and R script (https://osf.io/v7jth/?view_only=20f78478777648049d4455a1315420ef).

## Materials

We conducted a pilot study to create the social–political opinion statements. For our stimuli, we aimed to have ambiguous statements as the illusory truth effect is strongest for such statements (Fazio et al., 2017). Moreover, we assessed whether participants were indeed able to identify the social–political opinion statements as an opinion. To do so, 51 participants were recruited from the United States of America via Amazon Mechanical Turk (*M*_age_ = 38.4, *SD*_age_ = 13.4, range = 24–74; 31 men, 20 women). The pilot study was a one-session study and all participants received financial compensation for their participation. The experiment was conducted online via Qualtrics. Data are available on OSF (https://osf.io/v7jth/?view_only=20f78478777648049d4455a1315420ef).

For the pilot study, we first created 50 statements derived from several social–political opinion polls from www.pewresearch.com of which 25 were more liberally and 25 more conservatively oriented. The syntax for social–political opinion statements (e.g., “The death penalty is one of the best deterrents for violent crime”) was similar to general opinion statements; however, they were characterized by information about controversial social–political events (e.g., abortion, death penalty, and social media) and contained subjective elements (e.g., “best”, “could”, “likely”). In the first part of the pilot study, we asked participants to provide a truth rating on a 7-point Likert (*1* = *not truthful, 7* = *very truthful*) scale about all the social–political opinion statements. We asked participants first to give their truth ratings to avoid the possibility of inflated truth ratings due to the illusory truth effect. Afterward, during Part 2, participants were again presented with all social–political opinion statements but also true information statements and asked them to indicate whether the statement is a fact or opinion based on its syntax structure (see Experiment 1 for exact instructions).

We identified 17 liberal social–political statements and 16 conservative social–political statements with truth ratings between 3.6 and 5.6 on a 7-point Likert scale. Moreover, the chosen statements were, on average, accurately encoded as an opinion with an accuracy of 80% or higher (see supplementary materials on OSF). We used these 33 social–political opinion statements as our stimuli in Experiment 2. Of these 33 statements, we chose 9 critical items and 9 control items a priori for Experiment 2. Truth ratings for the 9 critical items (*M* = 4.60, *SD* = 0.71) did not statistically significantly differ from the 9 control items (*M* = 4.59, *SD* = 0.72), *t* (50) = 0.11, *p* = 0.91, Cohen’s *d*_*z*_ = 0.02, 95% CI [− 0.26, 0.29].

All other materials were the exact same as in Experiment 1 except that the misinformation statements were replaced by the social–political opinion statements. Hence, we used the same 33 true information and general opinion statements used in Experiment 1. As in Experiment 1, we selected 27 critical items a priori that were repeated and used for the data analysis. The critical items consisted of nine true information, nine general opinion, and nine social–political opinion statements (see Appendix B and the preregistration). We also a priori selected 27 control items that were not repeated and only shown during Part 2. The control items were only presented during Part 2 of the experiment and consisted of nine true information, nine general opinion, and nine social–political opinion statements. The same two attention checks were also used in Experiment 1.

## Procedure and design

Design and procedure were exactly the same as in Experiment 1, except that participants were presented with social–political opinion statements instead of misinformation statements.

## Results

### Manipulation and attention checks

During Part 1, participants correctly encoded, on average, 89% of the true information statements as a fact, 74% of general opinion statements as an opinion, and 85% of social–political opinions statements as opinions. Taken together, these findings suggest that participants generally encoded the information as intended, although it seemed that general opinion statements were more difficult to identify as an opinion based on their syntax structure. However, we also performed statistical analyses only considering items that were correctly encoded as a fact or opinion during Part 1 of the study.

One participant failed both attention check and the participants’ data was excluded from the data analyses. One participant failed the first attention check and eleven participants failed the second attention check.^9^

## Confirmatory analyses

### Repeated vs new statements

*True Information*. A one-tailed paired-sample t test showed that repeated true information statements received higher subjective truth ratings (*M* = 5.71, *SD* = 1.09) compared with new true information statements (*M* = 5.55, *SD* = 1.14), *t* (184) = 4.21, *p* < 0.001, Cohen’s *d*_*z*_ = 0.31, 95%CI [0.19, ∞] (see Table 2). Additionally, the TOST procedure with raw equivalence bounds Δ_*L*_ = − 0.20 and Δ_*U*_ = 0.20 showed a statistically significant result against the Δ_*L*_, *t* (184) = 9.69, *p* < 0.001, but did not find a statistically significant result against the Δ_*U*_, *t* (184) = − 1.27, *p* = 0.10. This indicates that the illusory truth effect for true information statements was statistically significant and was not statistically equivalent.

**Table 2:** Descriptive Statistics of Subjective Truth Ratings for each Type of Statement of Experiment 2

Type of statement	*N*	*M*	*SD*
True information			
Repeated	185	5.71	1.09
Repeated–correctly encoded	185	5.74	1.13
New–control	185	5.55	1.14
General opinion			
Repeated	185	4.39	0.99
Repeated–correctly encoded	183	4.22	1.05
New–control	185	4.46	0.99
Social–political opinion			
Repeated	185	4.18	1.05
Repeated–correctly encoded	181	4.11	1.09
New–control	185	4.14	0.98

*N* stands for sample size,* M* stands for mean, *SD* stands for standard deviation. Repeated statements were the 9 critical items chosen a priori that were presented during Part 1 and therefore repeated in Part 2. Repeated–Correctly Encoded statements included only critical items that were correctly encoded as a “fact” or “opinion” during Part 1. New – Control statements were control items that were only presented once during Part 2. Two participants did not correctly encode any general opinion statement as an opinion and four participants did not encode any social–political opinion as an opinion and were removed from the analyses. Removing the participants did not alter the pattern of results

*General Opinion*. A two-tailed paired-sample t test showed that there were no statistically significant differences between truth ratings for repeated general opinion statements (*M* = 4.39, *SD* = 0.99) and new general opinion statements (*M* = 4.46, *SD* = 0.99), *t* (184) = − 1.42, *p* = 0.16, Cohen’s *d*_*z*_ = − 0.08, 95% CI [− 0.23, 0.06]. Moreover, the TOST procedure with equivalence bounds Δ_*L*_ = − 0.20 and Δ_*U*_ = 0.20 showed statistically significant results against the Δ_*L*_, *t* (184) = 2.85, *p* = 0.002, and against the Δ_*U*_, *t* (184) = − 5.69, *p* < 0.001. This indicates that repeated general opinion statements did not receive statistically significantly higher subjective truth rating compared with new general opinion statements and this was statistically equivalent.

*Social–Political Opinion*. A two-tailed paired-sample t test did not find a statistically significant difference between subjective truth rating for social–political opinion statements that were repeated (*M* = 4.18, *SD* = 1.05) and new statements (*M* = 4.14, *SD* = 0.98), *t* (184) = 0.60, *p* = 0.55, Cohen’s *d*_*z*_ = 0.04, 95% CI [− 0.10, 0.19]. Additionally, results from the TOST procedure with equivalence bounds Δ_*L*_ = − 0.20 and Δ_*U*_ = 0.20 showed statistically significant results against the Δ_*L*_, *t* (184) = 3.87, *p* < 0.001 and against the Δ_*U*_, *t* (184) = − 2.67, *p* = 0.004. These results show that repeated social–political opinion statements did not receive statistically significantly higher subjective truth rating compared with new social–political opinion statements and this was statistically equivalent.

### Enhanced illusory truth effect

In contrast to Experiment 1, we did not find evidence that the illusory truth effect was stronger for true information statements correctly encoded as a fact (*M* = 0.19, *SD* = 0.65) compared with true information statements in general (*M* = 0.15, *SD* = 0.50),* t* (184) = 1.25, *p* = 0.11, Cohen’s *d*_*z*_ = 0.09, 95% CI [− 0.03, ∞]. Equivalence tests using the TOST procedure with raw equivalence bounds Δ_*L*_ = − 0.20 and Δ_*U*_ = 0.20 showed a statistically significant result against the Δ_*L*_, *t* (184) = 7.82, *p* < 0.001, and against the Δ_*U*_, *t* (184) = − 5.33, *p* < 0.001. This indicates that correctly encoded true information statements did not receive statistically significantly higher subjective truth ratings than new true information statement and this was statistically equivalent.

### Illusory truth effect and encoding

As in Experiment 1, we examined the illusory truth effect for each type of statement when only considering statements that were correctly encoded as a fact or opinion based on its syntax structure.

*True Information*. A one-tailed paired-sample *t* test showed that repeated true information statements received higher subjective truth ratings (*M* = 5.74, *SD* = 1.13) compared with new true information statements (*M* = 5.55, *SD* = 1.14), *t* (184) = 4.02, *p* < 0.001, Cohen’s *d*_*z*_ = 0.30, 95% CI [0.17, ∞] (see Table 2). Additionally, the TOST procedure with raw equivalence bounds Δ_*L*_ = − 0.20 and Δ_*U*_ = 0.20 showed a statistically significant result against the Δ_*L*_, *t* (184) = 8.22, *p* < 0.001, but did not find a statistically significant result against the Δ_*U*_, *t* (184) = -0.18, *p* = 0.43. These results show that the illusory truth effect for true information statements was statistically significant and not statistically equivalent.

*General Opinion*. Two participants did not correctly encode any of the general opinion statements as an opinion and were removed from the analysis. A one-tailed paired-sample t test showed that repeated general opinion statements (*M* = 4.22, *SD* = 1.05) received lower subjective truth ratings than new general opinion statements (*M* = 4.46, *SD* = 0.99), *t* (182) = − 3.61, *p* < 0.001, Cohen’s *d*_*z*_ = − 0.27, 95% CI [-∞, − 0.14]. Moreover, the TOST procedure with equivalence bounds Δ_*L*_ = -0.20 and Δ_*U*_ = 0.20 did not show a statistically significant result against the Δ_*L*_, *t* (182) = − 0.28, *p* = 0.61, but did find a statistically significant result against the Δ_*U*_, *t* (182) = − 6.98, *p* < 0.001. These results indicate that the reversed illusory truth effect for general opinions was statistically significant and not statistically equivalent.

*Social–Political Opinion*. Four participants did not correctly encode any of the social–political opinion statements as an opinion and were removed from the analysis. A one-tailed paired-sample t test did not find a statistically significant difference between subjective truth rating for social–political opinion statements that were repeated (*M* = 4.11, *SD* = 1.09) compared with new statements (*M* = 4.14, *SD* = 0.98), *t* (182) = − 0.19, *p* = 0.42, Cohen’s *d*_*z*_ = − 0.01, 95% CI [-∞, 0.11]. Additionally, results from the TOST procedure with equivalence bounds Δ_*L*_ = − 0.20 and Δ_*U*_ = 0.20 showed statistically significant results against the Δ_*L*_, *t* (182) = 2.69, *p* = 0.004 and against the Δ_*U*_, *t* (182) = − 3.09, *p* = 0.001. These results indicate that the reversed illusory truth effect for social–political opinions were not statistically significant and not statistically equivalent.

## Discussion

The results of Experiment 2 showed that repeated true information statements were perceived as more truthful than new true information statements (i.e., illusory truth effect). However, as in Experiment 1 and in line with our predictions, we did not find the illusory truth effect for general opinion statements. Also, as observed in Experiment 1, we found evidence of a reversed illusory truth effect when only considering general opinion statements that were correctly encoded as an opinion during Part 1 of the experiment.

In contrast to the conclusions of Arkes and colleagues (1989), we did not find evidence that repeating social–political opinion statements increased the subjective truth ratings compared with new statements. However, in the study by Arkes and Colleagues (1989), participants did not initially classify the social–political opinions as an opinion. It is possible, in line with the referential theory (Unkelbach & Rom, 2017), that the act of encoding opinion statements as an opinion served as a cue that elements within a statement are incoherent. Perhaps, this perceived incoherency impaired the processing fluency which could have led to the absence of the illusory truth effect for general and social–political opinion statements.

However, because participants initially encoded the statements as an opinion or a fact in Experiment 1 and 2, an empirical question remained whether the absence of the illusory truth effect is caused by the stimuli itself (i.e., opinion statements) or by the initial encoding (i.e., classifying it as an opinion). Hence, in Experiment 3, we isolated that variable by scrutinizing whether the stimuli itself or the encoding undermined the illusory truth effect. To do so, we used the same stimuli and a similar procedure as in Experiment 2. The primary difference was that during the encoding phase, participants were not instructed to indicate whether the presented statement was a fact or an opinion based on its syntax structure, but were instead told to assign each statement to a topic category. Henderson and colleagues (2021) used this same methodological approach as an encoding manipulation for the illusory truth effect. We expected to once again observe the effect for true information statements (Brashier & Marsh, 2020b). Moreover, based on the referential theory of Unkelbach and Rom (2017), we expected that because participants were not explicitly instructed to encode the relatively unknown and plausible general and social–political opinion statements as an opinion, they would not perceive the elements within such statements as incoherent. Thus, we expected to observe an illusory truth effect for general and social–political opinion statements, when not encoded as such.

## Experiment 3

Experiment 3 was identical to Experiment 2 except that during part 1 of the experiment, participants assigned each statement to a topic category, instead of indicating whether it was a fact or opinion based on its syntax structure.

## Methods

### Participants

We used the same a priori power analysis as in Experiments 1 and 2. Thus, we needed 180 participants. In total, we recruited 189 participants from Amazon Mechanical Turk. MTurk participants were recruited by posting a hit named “How do we judge statements?” The requirements for MTurk participants were a HIT approval rate of 98% and more than 5000 HITs approved. As in Experiments 1 and 2, we had two attention checks in Part 1 and Part 2 of the experiment. Three participants failed both attention checks so the data of this participant for the data analysis was excluded. Hence, we had data from 186 participants for the statistical analyses (*M*_age_ = 41.6, *SD*_age_ = 11.5, range = 19–70; 83 men, 103 women). Participants took on average 27.0 min (*SD* = 10.2; range = 12.1–71.4) to complete the study. All participants were financially compensated $2.00 for participation, regardless of whether they correctly answered the attention checks.

The Institutional Review Board of Grand View University approved the study. Moreover, we preregistered Experiment 3 on the OSF ( https://osf.io/9vwx4/?view_only=477c322c49c5474594feb55d3dbfbb02), and uploaded the data and R script (https://osf.io/ukhb7/?view_only=9843b3f06c0649ebab20d84e8387268e).

## Materials

We used the same materials as in Experiment 2, except for the first attention check which we adapted to be in line with the topic categories (i.e., The present city of Atlanta was originally named Terminus (To show that you have read this information, we would like you to answer “Sports” on this page).

### Design and procedure

We used a within-subject design. After participants gave their informed consent to participate in the study, they received the following instructions:

*“Previous research has revealed that high school students are not always able to indicate the correct topic category of particular statements. In this study, we are interested whether you are able to indicate to which topic category a statement belongs. The topic categories you can choose for each statement are: (1) Art & Entertainment, (2) Geography, (3) History & Politics, (4) Language, (5) Science, Nature & Technology, and (6) Sports. Hence, in the next phase you will receive multiple statements and then we would like you to indicate to which topic category the statement belongs.”*

We instructed participants to assign each statement to a topic category to keep the procedure of Experiment similar to Experiments 1 and 2. Moreover, using this procedure, we were able to ascertain that participants read the statements, and we avoided participants attempting to give consistent truth ratings during part 2 of the experiment if they were asked to initially rate the truthfulness during the encoding phase (Nadarevic & Erdfelder, 2014).

Afterward, participants completed in a 5-min filler task (i.e., playing Tetris). Then, as in Experiments 1 and 2, participants were given a list of 63 statements (27 critical repeated items, 27 control/new items, 9 distractor/new items) and were asked to indicate the truthfulness of each statement on a 7-point Likert scale (*1* = *not truthful, 7* = *very truthful*). Afterward, participants were thanked and debriefed.

## Results

### Confirmatory analyses

#### Repeated vs new statements

*True information.* A one-tailed paired-sample t test showed that repeated true information statements received higher subjective truth ratings (*M* = 5.11, *SD* = 0.97) compared with new true information statements (*M* = 4.99, *SD* = 0.93), *t* (185) = 2.09, *p* = 0.02, Cohen’s *d*_*z*_ = 0.15, 95% CI [0.03, ∞] (see Table 3). Additionally, the TOST procedure with raw equivalence bounds Δ_*L*_ = − 0.20 and Δ_*U*_ = 0.20 showed a statistically significant result against the Δ_*L*_, *t* (185) = 5.42, *p* < 0.001, but did not find a statistically significant result against the Δ_*U*_, *t* (185) = − 1.24, *p* = 0.11.

**Table 3:** Descriptive Statistics of Subjective Truth Ratings for each Type of Statement of Experiment 3

Type of statement	*N*	*M*	*SD*
True information			
Repeated	186	5.11	0.97
New – control	186	4.99	0.93
General opinion			
Repeated	186	4.79	0.96
New – control	186	4.67	0.95
Social–political opinion			
Repeated	186	4.83	0.93
New–control	186	4.71	0.92

*N* stands for sample size,* M* stands for mean, *SD* stands for standard deviation. Repeated statements were the 9 critical items chosen a priori that were presented during Part 1 and therefore repeated in Part 2. New – Control statements were control items that were only presented once during Part 2

*General Opinion*. A one-tailed paired-sample t test showed that repeated general opinion statements had higher subjective truth ratings (*M* = 4.79, *SD* = 0.96), than new general opinion statements (*M* = 4.67, *SD* = 0.95), *t* (185) = 2.26, *p* = 0.01, Cohen’s *d*_*z*_ = 0.17, 95% CI [0.04, ∞]. Moreover, the TOST procedure with raw equivalence bounds Δ_*L*_ = − 0.20 and Δ_*U*_ = 0.20 revealed a statistically significant result against the Δ_*L*_, *t* (185) = 5.86, *p* < 0.001, but did not find a statistically significant result against the Δ_*U*_, *t* (185) = − 1.34, *p* = 0.09.

*Social–Political Opinion*. A one-tailed paired-sample t test indicated that repeated social–political statements received higher subjective truth rating (*M* = 4.83, *SD* = 0.93) compared with new social–political opinion statements (*M* = 4.71, *SD* = 0.92), *t* (185) = 1.99, *p* = 0.02, Cohen’s *d*_*z*_ = 0.15, 95% CI [0.02, ∞]. Equivalence tests using the TOST procedure with raw equivalence bounds Δ_*L*_ = − 0.20 and Δ_*U*_ = 0.20 showed a statistically significant result against the Δ_*L*_, *t* (185) = 5.29, *p* < 0.001, but did not find a statistically significant result against the Δ_*U*_, *t* (185) = − 1.31, *p* = 0.10.

## Exploratory analyses

*Effect of Encoding*. We examined whether the encoding method affected the subjective truth ratings. More specifically, we examined whether true information statements that were encoded as a fact (Exp. 2) received higher subjective truth ratings than when such statements were encoded based on topic categories (Exp. 3). Moreover, we also examined whether general or social–political opinion statements received lower subjective truth ratings when they were encoded as an opinion (Exp. 2) compared with as a topic category (Exp. 3). Such integrative data analysis can have many advantages (e.g., increased statistical power) and because our study designs, materials, and procedures were rather similar, we deemed such data analysis appropriate for exploratory purposes (for an overview of integrative data analysis, see Curran & Hussong, 2009). However, one potential drawback is the lack of randomization and therefor results should be interpreted with caution.

*True Information***.** A two-tailed Welch independent sample t test showed that true information statements that were encoded as a fact received higher subjective truth ratings (*M* = 5.74, *SD* = 1.13) than true information statements encoded as a topic category (*M* = 5.11, *SD* = 0.97), *t* (360.44) = 5.73, *p* < 0.001, Cohen’s *d* = 0.60, 95% CI [0.39, 0.81].

*General Opinion*. A two-tailed Welch independent sample t test revealed that general opinion statements that were encoded as an opinion received lower subjective truth ratings (*M* = 4.22, *SD* = 1.05) compared with general opinion statements encoded as a topic category (*M* = 4.79, *SD* = 0.96), *t* (362.58) = − 5.44, *p* < 0.001, Cohen’s *d* = − 0.57, 95% CI [− 0.78, − 0.36].

*Social–Political Opinion*. A two-tailed Welch independent sample t test showed that social–political opinion statements that were encoded as an opinion received lower subjective truth ratings (*M* = 4.11, *SD* = 1.09) compared with social–political opinion statements encoded as a topic category (*M* = 4.83, *SD* = 0.93), *t* (356.38) = − 6.85, *p* < 0.001, Cohen’s *d* = − 0.73, 95%CI [− 0.94, − 0.51].

## Discussion

In Experiment 3, we found the illusory truth effect for true information, general opinion, and social–political opinion statements. That is, subjective truth ratings were higher for repeated statements compared with new statements, irrespective of the type of information, when participants encoded the stimuli by simply assigning each statement to a topic category. This is in support of Arkes and colleagues (1989) who indeed argued that the illusory truth effect would also be observed for social–political opinion statements. However, when comparing the results of Experiment 2 and 3, our results suggest that how information is encoded plays a crucial role in the illusory truth effect. More specifically, true information statements received higher subjective truth ratings when encoded as a fact instead of when assigned a topic category, while general opinion, and social–political opinion statements received lower subjective truth ratings when they were encoded as an opinion (vs. assigning topic category). Hence, it seems that if opinions are encoded as such, it can protect against the illusory truth effect.

## General discussion

Previous research has demonstrated that repeating information, true or false, is perceived as more truthful compared with new information, also known as the illusory truth effect (Brashier & Marsh, 2020b). Across three experiments, we examined whether such effects are also observed for general and/or social–political opinion statements. To do so, participants were presented with a list of true information, misinformation, general opinion and/or social–political opinion statements and were instructed to either indicate whether it was a fact or opinion based on the syntax structure (Exp. 1 & 2) or assign each statement to a topic category (Exp. 3). Afterward, participants rated the truthfulness of new and repeated statements.

In line with our prediction, we found evidence for the typical illusory truth effect in all three experiments (Dechêne et al., 2010). Specifically, we found that repeated true information (Exp. 1–3) and misinformation (Exp. 1) statements were perceived as being more truthful compared with new statements. This highlights the robustness of the illusory truth effect and shows it can be observed even with short delays between repetition (Henderson et al., 2021) and with different procedures. One explanation for the observed illusory truth effect can be derived from the referential theory of Unkelbach and Rom (2017) which postulates that coherency between elements in a statement is indicative of its fluency and, in turn, subjective truth. In Experiments 1 and 2, participants encoded true information and misinformation statements and had to indicate whether it was a fact based on its syntax structure. Our exploratory analyses showed that the initial classification of statements as a fact, led to higher subjective truth ratings (Exp. 2) compared when information was encoded via assigning topic categories (Exp. 3). It is possible that participants deemed information encoded as a fact more credible, increasing the coherency and/or processing fluency between elements within such statements resulting in higher subjective truth ratings. Previous research (Begg et al., 1992) has indeed shown that credibility of the source of information can affect the illusory truth effect. Moreover, our exploratory analyses of Experiment 1 showed that when only taking into account true information and misinformation statements correctly encoded as a fact, the illusory truth effect was enhanced. This suggests that how information is perceived and encoded might underpin the illusory truth effect. Even though we did not replicate this enhanced illusory truth effect in Experiment 2 for true information statements, we did find that information encoded as a fact led to higher subjective truth ratings compared with statements that were not (Exp. 2 vs Exp. 3). Hence, our results indicate that how information is encoded can increase the typical illusory truth effect.

## The Illusory Truth Effect on Opinions

 Interestingly, in line with our predictions and the conclusions of Arkes and colleagues (1989), we found the illusory truth effect for general opinion and social–political opinion statements when they were more shallowly encoded (i.e., assigning topic categories; Exp. 3). In other words, when using a typical procedure wherein opinion statements are simply repeated, it seems that, as observed with true information and misinformation statements, repetition led to higher subjective truth ratings compared with new statements. One explanation is that because participants were not explicitly instructed to encode information as a fact or opinion in Experiment 3, they were not cognizant that the statements were opinions. In line with both the processing fluency account (Reber & Schwarz, 1999) and referential theory (Unkelbach & Rom, 2017), when relatively unknown but plausible information is repeated, even when such statements are opinions, the perceived coherency between elements and processing fluency are increased leading to higher subjective truth ratings.

An alternative explanation for the observed illusory truth effect for general and social–political opinions in Experiment 3 can be that categorizing statements on topic categories did not induce an evaluative mindset. An evaluative mindset can be regarded as engaged thinking to validate and interpret presented information (Mayo, 2015). That is, even though participants in all experiments processed the words semantically, participants in the first two experiments had to separate a fact from an opinion which might have elicited such an evaluative mindset. Interestingly, previous research has shown that an evaluative mindset can reduce the illusory truth effect (Salovich et al., 2022). Hence, it is possible that prompting participants to carefully consider the presented information by, for example, judging whether the information is based on factual or subjective information can protect them against the illusory truth effect.

However, one of the most notable findings was that how opinion statements were encoded played a crucial role in whether the illusory truth effect was observed. That is, we did not observe the illusory truth effect for general opinion (Exp. 1–3) and social–political opinion (Exp. 2 & 3) statements when encoded as such. We actually found evidence of a reversed illusory truth effect for general opinion statements when encoded as such. That is, repeated general opinion statements that were correctly encoded as an opinion received lower subjective truth ratings as compared with new statements, indicating a boundary condition of the illusory truth effect. Furthermore, our results indicated that when opinion statements were encoded as an opinion, they received lower subjective truth ratings (Exp. 2) than when they were encoded in a more shallow manner (Exp. 3). This finding is in line with previous research examining the role of how information is encoded in the illusory truth effect. Specifically, it has been demonstrated that the illusory truth effect is reduced when information is processed more deeply (i.e., truth evaluation task) compared with shallowly processed information (i.e., comprehension task; Hawkins & Toch, 1992) or when participants are warned before encoding that half of the information they will be exposed to might be false (Jalbert et al., 2020). Moreover, it seems that when information is worded as a question (versus a statement) the illusory truth effect is eliminated (Calvillo & Harris, 2022). Additionally, the illusory truth effect is reversed when participants are instructed before encoding that they will have to judge later on whether the presented statements are true or false (Corneille et al., 2020; Exp. 3). Taken together, this suggests that how information is encoded might alter the illusory truth effect.

In accordance with the referential theory (Unkelbach & Rom, 2017), one potential explanation can be that the initial encoding of opinion statements as an opinion exposed that the elements within a given statement were incoherent. For instance, opinions oftentimes contain specific information or words, such as “best”, “worst”, and “should”, indicating its subjectivity which could indicate low accuracy and might lower the perceived coherency. Interestingly, Brashier and colleagues (2020a), showed that instructing participants to act like “fact checkers” and rate the accuracy of statements during encoding, protected against the illusory truth effect. Similar findings were observed when information originated from an unreliable source (Begg et al., 1992, experiment 4). Using this logic, our results imply that indicating during the encoding phase whether general opinion or social–political opinion statements were an opinion, indirectly pointed out its inaccuracy leading to lower subjective truth ratings for such statements.

Interestingly, the illusory truth effect for general opinion and social–political opinion statements when they are encoded as such can also be accounted for by the fluency processing account (Brashier & Marsh, 2020b). The fluency processing account posits that information is judged more truthful when the information is processed more fluently, wherein repetition can increase such processing fluency. This stems from the idea that people, on average, are more frequently exposed to the one truthful version (e.g., “The soccer World Cup Trophy was first called the Jules Rimet Trophy”) than several other alternative false versions (e.g., “The soccer World Cup Trophy was first called the Diego Maradona/Pelé/Franz Beckenbauer Trophy”). This notion does not apply to opinions wherein there is no one single version of the truth. In other words, people encounter different opinions on a daily basis which might serve as an indicator that the information is not trustworthy. Our results of Experiment 1 and 2 showed that processing fluency was only affected when participants encoded the opinion statements as an opinion. However, when general opinion and social–political opinion statements are simply repeated without explicitly or intentionally processing its veracity (Exp. 3), participants might have processed it as truthful information, leading to the typical illusory truth effect. It is possible that the initial encoding of information as an opinion decreases the processing fluency as it highlights the subjective information of a statement casting doubt on its veracity. However, when the specific evaluation of such statements is lacking, the mere repetition of these relatively unknown statements can lead to the illusory truth effect (Salovich et al., 2022). Hence, it seems that when repeated opinion statements are encoded as an opinion, it impairs the processing fluency and, in turn, no increase in subjective truth is observed in comparison with new general opinion statements.

An alternative explanation for the absence of this effect for opinion statements might be that people generally believe that their opinions are shared and for that reason difficult to change (Flynn et al., 2017; Leviston et al., 2013; Lewandowsky et al., 2017). That is, previous research has demonstrated that people resist changing their opinions, even after it has been debunked or corrected, also known as the continued influence effect (CIE; Johnson & Seifert, 1994). Moreover, sometimes the corrections or debunking can yield the opposite effect wherein people believe their misconceptions even more (Nyhan & Reifler, 2010; but see Kan et al., 2021). In our study, we found a similar type of resistance against changing opinion statements as participants did not increase or reduce their subjective truth ratings for repeated statements, when they encoded them as an opinion.

## Limitations and future research

There are some limitations of the current study that need to be addressed. To avoid ordering or sequence effects, we presented the statements completely at random for each participant. Because this was one of the first experiments examining the illusory truth effect on opinions, we opted for the most straightforward comparison and did not counterbalance the repeated and new statements. One potential issue is that the repeated statements were more believable than those used in the new condition. However, we used relatively unknown but plausible statements (Hassan & Barber, 2021) and chose the repeated and control items completely at random. Moreover, in our pilot study for Experiment 2, we chose items that received comparable truth ratings for repeated and control items and the results were similar to the other two experiments. Future research could further scrutinize the illusory truth effect for various opinion statements while controlling for potential confounds by counterbalancing the repeated and control statements.

Another possible limitation is the variety of completion times between participants for the three experiments. The majority of participants took, on average, 23.4, 24.4, and 27.0 min to complete Experiments 1, 2, and 3, respectively. However, some participants completed the experiments substantially faster or slower. This might suggest that some participants were not attentive. However, we included two attention checks in line with best practice recommendations (Abbey & Meloy, 2017; Thomas & Clifford, 2017) to exclude inattentive participants. Even with the inclusion of the participants who failed the attention checks, the pattern of results across the three experiments did not change. Moreover, we conducted several additional exploratory analyses to see whether duration time was correlated with the illusory truth effect and found limited to no evidence for statistically significant correlations (see additional analyses on OSF). It is possible that some participants initiated the experiment but did not immediately start, possibly leading to the varying completion times. Taken together, this suggests that the varying completion times did not alter the observed results in our experiments.

Our results provide evidence of the typical illusory truth effect, but highlights that how information is encoded might alter the effect. In line with the recommendations of Henderson and colleagues (2021), we showed that simply repeating information can lead to higher truth rating (versus new information) for true information, misinformation, general opinion, and social–political opinion statements, providing evidence that the illusory truth effect might be generalizable across stimuli. However, it is important to note that we used a Western, Educated, Industrialized, Rich, and Democratic (WEIRD) sample and future research could scrutinize whether our results generalize to non-WEIRD societies. Further, in our study, participants participated online wherein statements were shown one-by-one with no additional information. Although the online setting of our experiments accurately reflects the environment on social media, it is quite different from, for example Twitter, where such information comes with the author which might have additional effects on people’s judgment of truth. Hence, future research could examine whether providing reliable or unreliable sources impacts the illusory truth effect for opinions. Lastly, the use of social–political opinions is heavily based on the current political climate and might not be relevant in future research. However, our results do indicate that the illusory truth effect can be observed for such social–political opinion statements, but that such an effect fades when it is indeed encoded as an opinion.

An interesting empirical question is whether the illusory truth effect is also not observed when people agree with the opinion statements. In other words, it might be that the illusory truth effect is detected when participants agree with the social–political opinion statements, as a type of confirmation bias (Nickerson, 1998). Alternatively, instead of measuring truthfulness for opinion statements, it could be that participants are more inclined to agree with repeated opinions compared with new opinions. That is, they might not indicate that they perceive repeated opinions as more truthful because of its inherit subjectivity, but do agree with them more. However, the main aim of the current experiments was to examine the illusory truth effect for opinions in general when encoded as such, and therefore did not take into account to what degree participants agreed with the social–political opinion statements. Future studies could assess whether the (reversed) illusory truth effect is observed when people agree with the social–political opinions. Moreover, it could be analyzed whether repeating opinion statements increases people’s agreement with the opinions instead of truthfulness.

## Practical implications

With the expanse of social media platforms, people have to constantly evaluate what information is trustworthy and what should be discarded. Our results showed that when information is repeated and the content is not scrutinized during encoding, it can lead participants to judge such information as subjectively more truthful than new information. However, in line with previous research (Calvillo & Harris, 2022; Corneille et al., 2020; Jalbert et al., 2020), our results showed that the way in which information is encoded plays a crucial role in the illusory truth effect. Specifically, we showed that a simple instruction to assess the information as a fact or opinion can eliminate or reverse the illusory truth effect for opinion statements. In combination with previous research (Calvillo & Harris, 2022; Corneille et al., 2020; Jalbert et al., 2020), this suggests that easy and direct prompts can help people actively process the source, veracity, and type of information. Interestingly, social media platforms have already introduced fact checks to combat the spread of misinformation and research indicates that such fact checks can indeed reduce the belief in misinformation (Brashier et al., 2021; Clayton et al., 2020). A possible addition could be to also communicate to the reader whether the presented information is an opinion.

## Conclusion

Taken together, we replicated the typical illusory truth effect for true information, misinformation, general opinion and social–political opinion statements and showed that the effect can be observed using a short delay between repetitions (Henderson et al., 2021) and different procedures. However, our results indicate a boundary condition of the illusory truth effect wherein the manner in which information is encoded can alter the effect. That is, when true information is encoded as a fact, it can lead to higher subjective truth ratings compared to statements that are encoded more shallowly via assigning topic categories. Alternatively, when general opinion and social–political opinion statements are encoded as such, it seems to protect against the illusory truth effect. In fact, we found a reversed illusory truth effect for general opinion statements when considering only the statements that were encoded correctly as an opinion. In line with previous research (Brashier et al., 2020a), we argue that prompting people to assess the veracity and/or type of information during encoding can alter the illusory truth effect, and sometimes even reverse it. That is, our results indicate that the illusory truth effect might be moderated by the manner in which encoded. Last, and fortunately, these findings suggest the perception of truth may not be as easily influenced by repeating an opinion when they are perceived as such. In that regard, it seems facts indeed outweigh opinions.

## Data Availability

The data and R scripts that support the findings of this study are openly available on the Open Science Framework at https://osf.io/u67kg/?view_only=20c8eeb39e884f81a4345629bcb4322d (Exp. 1), https://osf.io/v7jth/?view_only=20f78478777648049d4455a1315420ef (Exp. 2), and https://osf.io/ukhb7/?view_only=9843b3f06c0649ebab20d84e8387268e (Exp. 3).

## Appendix A

**Table:**

All 99 true information, misinformation, and general opinions			
	True Information	Misinformation	General Opinions
1	The first Hall of Fame was in New York University*	A gond is another name for a poodle*	Lake Erie is the shallowest and, therefore, least enjoyable of the great lakes*
2	One carat, used to weigh stones, equals exactly 200 mg*	The gestation period of a giraffe is 1425 days*	Midway Island is not part of the state of Hawaii but has the greatest beaches*
3	Rogun Dam in Russia is the highest dam in the world*	The longest fangs of any snake are those of the rattlesnake*	The floppy disk was the least convenient way to store data*
4	There are currently 5 known ‘dwarf planets’ in our solar system*	Gunpowder is a mixture of saltpeter, sugar, and charcoal*	The Gobi desert is in both China and Mongolia making it a wonderful place to visit*
5	The White House stands on 18 acres of land*	Earth’s moon is the largest moon in our solar system*	The tallest and scariest active volcano is the Ojos del Salado*
6	The odds of having 2-pair in poker is 20 to 1*	The largest island in a lake is Manitoulin in Nebraska*	Wool is the traditional gift for a seventh anniversary and disliked by most who follow the tradition*
7	Hippopotamus can run faster than humans at 30 km per hour*	The saltiest lake in the world is the Don Juan lake in Africa*	Maurice Garin was the first winner of the Tour de France and was the best athlete of his time*
8	The diameter of the moon is 2,160 miles*	There are 100 active volcanoes in the world*	Winston Churchill coining the phrase “Iron Curtain” was the most significant moment of the Cold War*
9	A Pilot whale is actually a kind of dolphin*	A stingray must be eighty years old before it can reproduce*	Napoleon, born on the island of Corsica, was one of the most terrifying conquerors in history*
10	The soccer World Cup Trophy was first called the Jules Rimet Trophy**	Al Capone was the founder of the John Birch society**	Instead of the kangaroo, the Tasmanian Devil is the most iconic animals in Australia**
11	The first cat show was in Great Britain's Crystal Palace in 1871**	Gold is associated with the 18th wedding anniversary**	Walter Hunt was a brilliant inventor who created the safety pin**
12	The Great Galaxy is the galaxy closest to the Milky Way**	Christmas Island, in the Indian Ocean, is a territory of America**	The bicycle is the most important invention for quick individual travel**
13	The first man to sail around the globe solo was Joshua Slocum**	A Jupiter day is 100 Earth days long**	Cinnamon trees smell wonderful and grow up to 20 m tall**
14	The eskimo pie was invented by Christian K. Nelson**	Walter Cronkite was born in Sydney, Australia**	Cleo, the goldfish in Pinnochio, is the most underappreciated character in that story**
15	The New Mexican whiptail lizard can reproduce without any male contact**	Originally known as "diastoid", malted milk was rebranded in 1987**	The refrigerator was the greatest invention of the 1700s**
16	The zipper was invented by Elias Howe in 1851**	Benjamin Franklin is known as the father of geometry**	William Gray patented the first pay phone in 1891 transforming public communication more so than the telegraph**
17	The monk dolphin is the only species of dolphin in its genus**	The Yen is the monetary unit of Bangladesh**	Manhouts who are keepers and drivers of elephants have a challenging but rewarding job**
18	The Karakum Desert's name means "black sand" in Turkic languages**	Fairy armadillos, found primarily in Florida, live entirely in trees**	Margaret Gorman, the first Miss America Pageant winner, was the most beautiful woman in the world in 1920s**
19	Nina Kuscsik was the first women's winner of the Boston Marathon**	The top speed of an elephant is 55 miles per hour**	Braille, based on a code of sixty-three characters, is easier to learn than most people realize**
20	The origin of loaded dice is from Egypt, dating back to 3000 BC**	The first American post office was founded in Honolulu, HI**	The invention of the typewriter transformed literacy and reading around the world**
21	Maria Goppert was the 2nd female Nobel Laureate in physics**	A group of hares is called a common**	The first MVP in professional baseball was Frank Frisch who was the greatest second basemen of all time**
22	A group of hippopotamuses is called a bloat***	Australia has the greatest number of people over the age of 80***	The United States Capitol is the most beautiful government buildings in the world***
23	The African baobab tree only opens its blossoms to moonlight***	Agate is a gem found in Las Vegas and Boston***	The Mauna Loa is the largest and most extraordinary volcano in the world***
24	The Dirham is the monetary unit of Morocco***	The first 3-D film made was The Wizard of Oz***	Sir Mark Brunel constructed the Thames Tunnel in London which was the best addition to British public transit***
25	Australian-American Nobel laureate Elizabeth Blackburn co-discovered telomerase***	A dorter is the monks' lavatory in a monastery***	Zosimus of Alexandria, the first known alchemist, had the most influence on chemistry today***
26	Speusippos was a Greek philosopher alive in the 4th Century BC***	The first gymnastics instruction at a college was offered at the University of Iowa***	A Bandar sounds funny, but is another name for the Rhesus monkey***
27	A decibel is a unit of measurement for sound intensity***	The Humason comet has an orbital period of 1 year***	The Mayflower carried 102 pilgrims who changed the course of history for the betterment of freedom***
28	The Danube is the second longest river in Europe***	A group of apes is called an apricot***	A group of porcupines, called a prickle, is actually a really cute sight***
29	The spiny anteater is a mammal that lays eggs***	The eraser tip was the invention of George Washington***	Folegandros is the most luxurious island in the Mediterranean ocean***
30	The Ural Mountain Range separates Asia and Europe***	Aconcagua is the smallest mountain in South America***	Cantaloupes are delicious and originated in a region from India to Africa***
31	The average longevity of a kangaroo is seven years****	A hand, used to measure a horse's height, is a foot high****	The Lincoln penny, the first US coin to carry a portrait, is an icon for coins around the world****
32	The bandicoot is a marsupial animal of Australia****	The rainiest region in the world is Atacama in Chile****	San Marino, the world's smallest republic, is located within Italy and is a great vacation destination****
33	The pan-American highway is the longest road in the world****	Charles de Gaulle airport is the largest airport in the world****	Peter Bruegel the Younger painted “The Crucifixion” and perfectly captured the historical nature of that moment****

* = critical items presented in Part 1 and Part 2. ** = distractor items presented in Part 1. *** = control/new items presented in Part 2. **** = distractor/new items presented in Part 2.

## Appendix B

**Table:**

All 99 true information, social–political opinions, and general opinions			
	True information	Social–Political opinions	General opinions
1	The first Hall of Fame was in New York University*	Our voter system should automatically register all eligible voters to eliminate obstacles to voting (Liberal)*	Lake Erie is the shallowest and, therefore, least enjoyable of the great lakes*
2	One carat, used to weigh stones, equals exactly 200 mg*	There should be federal laws about the fuel efficiency for cars (Liberal)*	Midway Island is not part of the state of Hawaii but has the greatest beaches*
3	Rogun Dam in Russia is the highest dam in the world*	Extreme patriotism is probably dangerous (Liberal)*	The floppy disk was the least convenient way to store data*
4	There are currently 5 known 'dwarf planets' in our solar system*	People who are undocumented should be given a path to citizenship instead of being deported (Liberal)*	The Gobi desert is in both China and Mongolia making it a wonderful place to visit*
5	The White House stands on 18 acres of land*	The death penalty is too cruel to be used as punishment in a civilized society (Liberal)*	The tallest and scariest active volcano is the Ojos del Salado*
6	The odds of having 2-pair in poker is 20 to 1*	All voters should be required to show a government-issued photo ID to vote (Conservative)*	Wool is the traditional gift for a seventh anniversary and disliked by most who follow the tradition*
7	Hippopotamus can run faster than humans at 30 km per hour*	The mainstream media is probably the cause of most problems in the United States of America (Conservative)*	Maurice Garin was the first winner of the Tour de France and was the best athlete of his time*
8	The diameter of the moon is 2,160 miles*	Spirituality is important to living a full and meaningful life (Conservative)*	Winston Churchill coining the phrase "Iron Curtain" was the most significant moment of the Cold War*
9	A Pilot whale is actually a kind of dolphin*	International trade deals are harmful to America because in most cases they lower wages for US workers (Conservative)*	Napoleon, born on the island of Corsica, was one of the most terrifying conquerors in history*
10	The soccer World Cup Trophy was first called the Jules Rimet Trophy**	Private drug company profits are probably why our healthcare is overpriced (Liberal)**	Instead of the kangaroo, the Tasmanian Devil is the most iconic animals in Australia**
11	The first cat show was in Great Britain's Crystal Palace in 1871**	Our education system should be one of the highest priorities in the federal budget (Liberal)**	Walter Hunt was a brilliant inventor who created the safety pin**
12	The Great Galaxy is the galaxy closest to the Milky Way**	Domestic terrorism from white nationalist groups should be taken more seriously (Liberal)**	The bicycle is the most important invention for quick individual travel**
13	The first man to sail around the globe solo was Joshua Slocum**	Election Day should be a national holiday (Liberal)**	Cinnamon trees smell wonderful and grow up to 20 m tall**
14	The eskimo pie was invented by Christian K. Nelson**	Welfare is an important safety net for people who just need some extra help (Liberal)**	Cleo, the goldfish in Pinnochio, is the most underappreciated character in that story**
15	The New Mexican whiptail lizard can reproduce without any male contact**	America’s openness to people from all over the world is essential to our identity as a nation (Liberal)**	The refrigerator was the greatest invention of the 1700s**
16	The zipper was invented by Elias Howe in 1851**	Abortion should be illegal except for cases of incest and rape (Conservative)**	William Gray patented the first pay phone in 1891 transforming public communication more so than the telegraph**
17	The monk dolphin is the only species of dolphin in its genus**	Police officers shouldn't be judged for making a mistake in a life-threatening situation (Conservative)**	Manhouts who are keepers and drivers of elephants have a challenging but rewarding job**
18	The Karakum Desert's name means "black sand" in Turkic languages**	If you don’t love the United States of America you should move somewhere else (Conservative)**	Margaret Gorman, the first Miss America Pageant winner, was the most beautiful woman in the world in 1920s**
19	Nina Kuscsik was the first women's winner of the Boston Marathon**	The prisoners in Guantanamo Bay are probably all guilty of something (Conservative)**	Braille, based on a code of sixty-three characters, is easier to learn than most people realize**
20	The origin of loaded dice is from Egypt, dating back to 3000 BC**	The death penalty is one of the best deterrents for violent crime (Conservative)**	The invention of the typewriter transformed literacy and reading around the world**
21	Maria Goppert was the 2nd female Nobel Laureate in physics**	Welfare is a terrible system for people who just don’t want to work hard (Conservative)**	The first MVP in professional baseball was Frank Frisch who was the greatest second basemen of all time**
22	A group of hippopotamuses is called a bloat***	Abortion is a very private issue and should be decided between the woman and her doctor (Liberal)***	The United States Capitol is the most beautiful government buildings in the world***
23	The African baobab tree only opens its blossoms to moonlight***	If someone says something racist you should confront and correct them (Liberal)***	The Mauna Loa is the largest and most extraordinary volcano in the world***
24	The Dirham is the monetary unit of Morocco***	Stricter gun control is the best way to stop mass shootings (Liberal)***	Sir Mark Brunel constructed the Thames Tunnel in London which was the best addition to British public transit***
25	Australian-American Nobel laureate Elizabeth Blackburn co-discovered telomerase***	Most reputable news organizations probably get most of the facts straight when reporting a story (Liberal)***	Zosimus of Alexandria, the first known alchemist, had the most influence on chemistry today***
26	Speusippos was a Greek philosopher alive in the 4th Century BC***	The U.S. is the greatest country in the world (Conservative)***	A Bandar sounds funny, but is another name for the Rhesus monkey***
27	A decibel is a unit of measurement for sound intensity***	People shouldn't be so easily offended by things others say (Conservative)***	The Mayflower carried 102 pilgrims who changed the course of history for the betterment of freedom***
28	The Danube is the second longest river in Europe***	If you came here illegally you should be deported as quickly as possible (Conservative)***	A group of porcupines, called a prickle, is actually a really cute sight***
29	The spiny anteater is a mammal that lays eggs***	Crime is best controlled using severe punishment as a deterrent (Conservative)***	Folegandros is the most luxurious island in the Mediterranean ocean***
30	The Ural Mountain Range separates Asia and Europe***	We shouldn’t teach children about LGBTQ issues because it ruins their innocence (Conservative)***	Cantaloupes are delicious and originated in a region from India to Africa***
31	The average longevity of a kangaroo is seven years****	The wealth gap is the one of the greatest problems in the United States of America (Liberal)****	The Lincoln penny, the first US coin to carry a portrait, is an icon for coins around the world****
32	The bandicoot is a marsupial animal of Australia****	The best way to decrease crime is prevention and rehabilitation (Liberal)****	San Marino, the world’s smallest republic, is located within Italy and is a great vacation destination****
33	The pan-American highway is the longest road in the world****	Taxing anyone, even the rich, is the worst way to stimulate the economy (Conservative)****	Peter Bruegel the Younger painted “The Crucifixion” and perfectly captured the historical nature of that moment****

* = critical items presented in Part 1 and Part 2. ** = distractor items presented in Part 1. *** = control/new items presented in Part 2. **** = distractor/new items presented in Part 2.

## Footnotes

1 Based on a forward reference search of the study by Arkes and colleagues (1989) via Google Scholar for articles published before the 3^rd^ of June, 2022. Articles were read completely and only included when they cited Arkes and colleagues (1989) as evidence in favor of the illusory truth effect in regards to opinions.

2 $$Cohen^{\prime}sd_{z} = \,\,\frac{meandifference}{{SD_{1}^{2} + SD_{2}^{2} - 2rSD_{1} \,\,SD_{2} \,}}\,\, = \,\,\,\frac{0.2}{{0.93_{1}^{2} + 0.96_{2}^{2} - 2 \times 0.5 \times 0.93 \times 0.96}}$$

3 To make sure that participants who failed one of the attention checks did not alter the results, we also ran the statistical analyses excluding all participants that failed at least one attention check. The pattern of results was similar and, therefore, we report the results as outlined in our preregistration. Moreover, exclusion of the participant that failed both attention checks did not alter the pattern of results.

4 Due to a technical error, the pre-registration can be found in the description section on OSF instead of the summary section.

5 We examined whether the use of fewer stimuli per type of stimuli led to varying standard deviations compared with research using more stimuli per class. Across the three experiments, we found comparable standard deviations for true information and opinion statements as in studies using more statements per class (e.g., Unkelbach & Speckmann, 2021).

6 Equivalence tests were not pre-registered for Study 1 but are reported alongside all analyses with the interpretations.

7 Three participants did not correctly identify any misinformation statement as a fact based on its syntax structure. It is possible they knew the information was incorrect and therefore did not want to indicate it as a fact. However, this remains speculative and, thus, we removed the data of these participants for this specific exploratory analysis. Removing the data of these participants did not alter the pattern of results.

8 We did not conduct this second exploratory analysis for general opinion statements as we did not find the illusory truth effect for such statements.

9 The pattern of results did not change when including the data of the participant that failed both attention checks. Moreover, we also checked whether including the data of participants that failed only 1 attention check would alter the pattern of results. We found that the pattern of results did not change.
